# Delivering the Nutritional Needs by Food to Food Fortification of Staples Using Underutilized Plant Species in Africa

**DOI:** 10.1155/2020/8826693

**Published:** 2020-12-22

**Authors:** Ernest Teye, Christabel Irene Deha, Rosemond Dadzie, Roseline Love MacArthur

**Affiliations:** ^1^University of Cape Coast, School of Agriculture, Department of Agricultural Engineering, Food Fraud and Product Integrity Research Group, Cape Coast, Ghana; ^2^University of Cape Coast, Faculty of Science & Technology Education, Department of Vocational and Technical Education, Cape Coast, Ghana

## Abstract

Sub-Saharan Africa (SSA) is among the poorest region in the world, and undernourishment continues to be a great challenge although this region is endowed with a lot of underutilized plant species (UUPS), which are rich in nutrients, especially micronutrients that are unavailable in staple foods. The potential for fortifying major staple foods with UUPS could be the remedy. This study seeks to provide an overview of the fortification of staple foods with UUPS in Africa and suggest the way forward for effective nutritional and health benefits. The review revealed that fortification of major staple foods has been investigated: maize with grain amaranth, soybean, and moringa; sweet potato with cowpea, sorghum, bambara groundnut, peanut, and moringa; cassava with African yam bean, breadfruit, pigeon pea, bambara groundnut, moringa, and cowpea; and sorghum with pearl millet and green peas. The others were yam with cowpea, plantain, and moringa, while rice was also fortified with baobab pulp and locust pulp. All these studies were found to be acceptable with dense nutritional properties. Specifically, micronutrients such as magnesium, phosphorous, zinc, potassium, and iron were increased while others showed rise in fibre and protein levels. The fortification of staple foods with UUPS has been shown to be promising; however, more designed feeding trials are required to verify the impact on reducing undernutrition and hidden hunger. To do this, it is recommended that rice fortified with UUPS should be targeted as rice is increasingly becoming the leading and important staple food in Africa.

## 1. Introduction

Nutritional deficiency is a major issue featuring prominently on the agenda of many developing countries and international partners, because it affects more than one-third of the global population and in sub-Saharan Africa (SSA), and one in four people remains undernourished [1–3]. This is attributed to the region being the poorest in the world [4]. The major deficient nutrients are vitamins and minerals, especially vitamin A, iodine, and zinc [5]. These deficiencies are the known cause of hidden hunger, which has staggering consequences on human health and well-being hampering economic productivity [6]. For instance, malnourished children cannot learn properly, appear stunted, and are underdeveloped with other adverse consequences. More so, it must be stated that the consequences of micronutrient deficiencies are not only limited to health parameters but have far greater impact on economies through secondary physical and mental challenges, and further alter productivity [5]. To overcome these challenges, several studies have shown that food fortification is one of the best strategies that are safe and effective in preventing micronutrient deficiencies. However, this approach is very expensive and not accessible to the rural propoor; it also focuses on biofortification and ignores sociocultural reasons that favour diets balanced by a variety of quality staple foods.

Underutilized plant species (UUPS) are plant species whose potential contribution to the national economy have not been adequately explored due to the decreased attention to their production, consumption, and utilization [7]. The Global Facilitation Unit for underutilized species (GFU) defines it as “those plant species whose potential are not fully exploited to contribute to food security and poverty alleviation.” UUPS are rich sources of nutrients and other health-related properties. They hold huge potential for reducing the impact of malnourishment and hidden hunger. The potential for their use in fortifying major staple foods in Africa is currently a novel approach and hold a brighter prospect for alleviating poverty and undernutrition. In addition, it improves the value of the underutilized crop and reduces postharvest losses. Africa is blessed with numerous types of rich underutilized plant species [8]. Conti and coworkers reported that there are numerous neglected and underutilized species known in sub-Saharan areas and categorized them under cereals, legumes, tubers, and leafy vegetables and concluded among other things that UUPS could represent an opportunity to enhance food security and empower women [8]. Despite the availability and nutritional benefits of these promising underutilized species, their use and incorporation into our everyday diet remain a challenge.

Therefore, the incorporation of these nutrient dense underutilized plant species could be the solution to the aforementioned dual challenges. Undernutrition and hidden hunger are manifested most in developing countries with a noticeable symptom of growth stunting, mostly among children, and impaired capacity to resist diseases [9]. Malnutrition can be defined as a clinical condition in which there is disequilibrium between nutrient intake and requirements [10]. Fortifying staple food with UUPS could be very essential in alleviating nutritional challenges as well as preventing the total extinction of these plant species.

Experts believe that fortification is one of the major public health interventions recommended to prevent and control micronutrient deficiencies [11]. However, the availability, accessibility, and acceptability of these fortificants remain a challenge in developing countries coupled with the poor sustainability of such an approach. On the other hand, the economic factors also hinder the total utilization for optimum health benefits.

These therefore call for an alternative measure that overcomes the challenges of the conventional approach of using chemical fortificants. The best option therefore could be the use of nutrient dense underutilized crops. Normally, these crops are climate adaptive, grow on marginal land with little or no agronomic care, and are already known by rural folks hence the ease of incorporating them into staple foods. Incorporating some amount of these nutrient dense underutilized crops into staple food is normally done to improve some essential nutrients. It especially holds a huge potential for developing countries. However, little or no review information is available on utilizing local nutrient dense underutilized plant species for fortifying staple food to deliver nutritional needs in curbing malnutrition. This study seeks to summarize information on fortification of staple foods using indigenous underutilized nutrient dense edible plant species and their strengths that is currently available, and suggest the way forward for effective nutritional and health benefits in Africa.

## 2. Nutritional Needs Situation in Africa

Nutritional needs if not met, lead to health-related challenges. In Africa, the nutritional situation is characterized by high rates of malnutrition and hidden hunger [12]. Hunger continues to be a huge problem in developing countries; Africa and Asia account for about 89 percent of the world's hungry people. For the purpose of this discussion, the focus will be on Africa. According to other researchers [13], the scale of food and nutrition challenges in Africa is staggering. To support this statement, the hunger and nutrition commitment index for Africa revealed that 58 million children under the age of five are stunted, with 13 million wasted and 10.3 million obese while 220 million are considered calorie deficient [14].

Among the nutritional deficiencies, micronutrient malnutrition, also known as hidden hunger, is the most serious problem. Large population in Africa is having nutritional problems such as protein energy deficiency (PED), iron deficiency anemia (IDA), iodine deficiency disorder (IDD), and vitamin A deficiency (VAD) [12]. On the other hand, currently, the most prevalent micronutrient deficiencies are vitamin A and iron [13]. This translates to about 163 million children and women of reproductive age being anaemic, while 44% of preschool children are vitamin A deficient with 24% of all child deaths attributable to vitamin A deficiency [13]. The other nutritional deficiency that is present but not alarming is obesity and its associated health problems such as cardiovascular diseases, cancer, and diabetes. It should be noted that in Africa, malnutrition affects all age groups from foetal stage to adulthood [12]; however, the most vulnerable groups are pregnant, lactating women, and young children given their high demand for nutrients [5, 15]. In addition, malnutrition in African is highly related to income levels and countries with low-income levels are having more chronic cases due to inadequate food consumption because food insecurity is determined by availability, accessibilty and utilization of food, and the stability of these three parameters [16].

## 3. Food to Food Fortification of Staple Food Using Underutilized Plant Species

### 3.1. Food Fortification

Food fortification is defined as the addition of one or more essential nutrients to a food, whether or not it is normally contained in the food, for the purpose of preventing or correcting a demonstrated deficiency of one or more nutrients in the population or specific population groups. On the other hand, WHO defines fortification as the practice of deliberately increasing the content of an essential micronutrient (vitamins and minerals, including trace elements) in food, to improve the nutritional quality of the food supply and provide a public health benefit with minimal risk to health. Fortification is effective if it uses staple foods as vehicles to deliver the required micronutrients that are generally lacking or not available in sufficient quantities in consumers' diet. This approach has been practiced since the early days of food fortification to target specific health conditions such as iodine deficiency through the iodization of salt, anaemia through the fortification of cereals with iron and vitamins, vitamin A-fortified margarine, and neural tube defects through the fortification of wheat flour with folic acid [5]. However, in SSA, the best alternative way of fortification could be the incorporation of nutrient dense edible plant species in staple foods. This is normally done by indigenous people with knowledge passed on by experienced relatives and subsequently resulted in recent advances in incorporating nutritious food materials into food staples. Therefore, food to food fortification would be more sustainable and an option to alleviate nutrient deficiencies in SSA as other authors have confirmed the health benefits of this approach [17, 18].

### 3.2. Justification for Food to Food Fortification

The justifications for food fortification are summarized by Allen and others [1], and Nkama and coworkers [19] also stated that fortification is done for the following reasons: to obtain the full complement of nutrients; to eradicate or eliminate nutritional deficiencies, people selecting foods with lower nutrient density, and local foods of known nutritional value with processed meals of unknown value; and provide diets for proper health.

Notwithstanding these important justifications, policy support requires location-specific background knowledge of dietary patterns as well as nutritional deficiency status to avoid potential excess intake and financial loss. Food to food fortification of staple food therefore provides an extra advantage of incorporating other food materials thereby reducing the pressure on relatively few food materials. This further supports food security by providing an alternative sources when the traditional food sources fail.

### 3.3. Advantage of Underutilized Plant Species

Underutilized plant species, also known as “Orphan” crops, are traditionally nutrient-rich plant species which are marginalized and are given little attention or are paid or are entirely ignored by agricultural researchers, plant breeders, and policymakers [20]. Their neglect and low utilization are partly due to lack of awareness of their economic and nutritional value and overemphasizing staple food such as rice, maize, yam, cassava, and recently orange flesh sweet potato. The advantages of these underutilized plant species are farfetched; they are rich in nutrients, adaptive to agroecological niches and marginal areas, and require little or no agriculture inputs [21]. These qualities could put underutilized plant species ahead in the fight against food insecurity in the face of climate change in Africa and even other parts of the world. Their enhanced use can bring about better nutrition as many underutilized plant species contain more vitamin C and provitamin A than widely available commercial species and varieties [21]. Underutilized plant species could therefore be significant in improving nutrition, generating income, maintaining ecosystem health, and empowering the poor and marginalized [20]. Therefore, focusing attention on the incorporation of these underutilized plant species into staple food would be an effective technique to help maintain a diverse and healthy diet and to combat nutritional deficiencies leading to zero “hidden hunger,” and other dietary deficiencies, particularly among the rural poor and the more vulnerable in developing countries.

### 3.4. Staple Food

Staple foods are normally eaten regularly to supply the body with energy and nutrients. The average African meal is made up of the majority of carbohydrates (46% cereals and 20% root and tuber) and a small amount (7%) of animal product [2, 3]. Furthermore, the world has over 50,000 edible plants, but rice, maize, and wheat are the major staple foods worldwide with about 50 worldwide calorie need [22]. Normally, these staple foods do not meet the total nutritional needs, and other variety of foods is required. However, in developing countries, the majority of rural people live on diet based on one or more of the staple foods that are mainly rich in carbohydrates. The staple foods mostly consumed in Africa are rice, maize, cassava, yam, and sweet potato. The others are taro, sorghum, plantain, and cocoyam. Their consumption has resulted in the vast majority suffering from “hidden hunger” and other malnourished-related diseases. Therefore, to help alleviate these challenges, there is the need to incorporate inexpensive nutrient-rich food sources into the already accepted staple food.

### 3.5. Food to Food Fortification of Staple Foods

Food to food fortification especially using underutilized nutrient dense plant species could provide a huge relief for malnutrition challenging the developing world. Numerous researchers have attempted to prove its potential as revealed in Table 1. Firstly, fortifying maize, a major staple food in Africa and the main condiment for many derived dishes, would be a major vehicle for reducing malnutrition. Kamotho and coworkers attempted biofortification of maize flour with grain amaranth and revealed that this enhanced the protein, iron, calcium, and zinc contents of the maize flour significantly and concluded that 40% gain amaranth gave the best result. However, 20% was the most acceptable blend by consumers [23]. This shows that using grain amaranth could provide a cheap and effective means of reducing hidden hunger such as iron and zinc deficiencies particularly among children. It will further be translated into improving healthy living and reducing neonatal and maternal death.

Sweet potato, another major staple food for African people, was fortified with cowpea and peanut at a percentage of 25% and 15%, respectively, and it was found acceptable with dense nutritional properties for infant weaning food, and subsequently, the functional properties of the flour were improved. Substitution of orange flesh sweet potato with Bambara groundnut in the formulation of a snack increased the magnesium, phosphorous, potassium, and iron contents in the composite snacks. The study showed that the development of OFSP substituted with Bambara groundnut up to 40% enhances the nutritional quality of the products and retains sensory properties with an acceptable consumer score, and with colour and sweetness being the main drivers [24].

Furthermore, composite flour made from local maize, banana, and soybean had high levels of potassium and sodium (19350 mg/kg and 12850 mg/kg, respectively) and appreciable levels of iron, zinc, and manganese. Physicochemical analyses showed total carbohydrate was relatively high in all the composite flour with a substantial amount of crude protein ranging from 5.46 ± 0.51 to 8.95 ± 0.51% and pH values from 6.13 ± 0.04 to 6.23 ± 0.01 [25]. The proximate results and mineral content made the banana composite flour an ideal product for weaning babies and infants. It must be emphasized that this further, could reduce postharvest losses of banana in Africa which at the moment is high [25].

Soybean, another emerging crop with a lot of governmental advertisements, has been shown to contain protein that has widely been used as a functional ingredient for food fortification [26]. In another study, the combination of oat bran, soya flour, and maize was good for obtaining high fibre and high-protein extrudate, but reduces the starch level in the mixture, which is undesirable due to the resulting increase in hardness [27]. The effect of soy flour fortification on the chemical and physicochemical properties of malted sorghum flour was also investigated [28], and the results show that the protein content of the flour mixes increased from 7.3% to 19.2%. The blend containing 40% soy flour substitution had the highest protein, fat, ash, and fibre contents. The mineral content increased while the antinutritional factors decreased as the soy flour substitution increases in the mixes. The functional properties of the flour decreased while the pasting viscosity and pH increased [28]; this further provides a great evidence for food to food fortification with great potential.

Tigernut-based beverage “Kunnu-aya” was fortified with underutilized *Vigna racemose* legume, and the proximate composition increased from 28 to 102%, 6 to 21%, and 2 to 11%, respectively, for protein, ash, and carbohydrate contents of the fortified food. However, a decrease in fat (6-22%) and crude fibre (12-5%) contents were observed. Furthermore, the mineral contents of the fortified “Kunnu-aya” increased significantly while the sensory attribute was more acceptable than the unfortified “Kunnu-aya” [29, 30].

Fortification of cereal-legume weaning food with orange flesh sweet potato (OFSP) was studied [31]. It was concluded that OFSP flour has the potential to be used at 25% replacement level in the soy-fortified roasted maize meal formulation, and OFSP is a useful ingredient with the potential to improve the *β*-carotene or vitamin A content. This will help alleviate vitamin A deficiency of children in Ghana and other countries with similar problems. Sweet potato fortified with fermented soybean flour in the formulation of cookies showed that 20% soybean flour was acceptable with high nutritious properties. Sweet potato-based infant flours fortified with soybean and sorghum flours showed promising results and concluded that the commercialization of sweet potato-based infant flours fortified with soybean and sorghum is recommended in the fight against malnourishment.

Furthermore, sweet potato flour fortified with avocado pear and turkey berry showed that majority of the proximate compositions were enhanced particularly, protein and fibre, and the vitamin C and mineral content were also improved. In addition to improving the mineral content [32], the authors concluded that fortifying sweet potato flour with avocado pear and turkey berry could be an easy and affordable means of improving rural nutrition, as it requires simple logistics for the ordinary rural household to prepare it.

Moringa oleifera leaf powder was used to fortify various dishes (porridge, ofam, beans and gari, waakye, apapransa, nkontomire sauce, and groundnut soup) in Ghana, and this increased the levels of Cu, Fe, Mn, Zn, and beta-carotene content of the diets. More so, these fortified diets were preferred by children and could be used as a vehicle for Moringa oleifera utilization for delivering micronutrients. It could be mentioned that food to food fortification using *M. oleifera* leaves has the potential of being a less expensive *β*-carotene and mineral source in the diets of many children in Ghana and other tropical countries where it is grown and could be easily adapted where children often have marginal vitamin A status [33].  Table 1 shows the fortification of some staple foods with underutilized plant species and their benefits. It could be seen that a lot has been attempted though there exist approximately 50,000 underutilized plant species. However, these findings could be very beneficial if what is done so far is incorporated into nutritional policies and implemented with governmental support and education. This would prove very useful in reducing malnutrition in developing counties.

## 4. Antinutrients

Antinutrients in plants are natural compounds that reduce the nutrient utilization or interfere with the absorption of nutrients, and they play a vital role in determining the use of plants for food [59]. Also, antinutrients influence the availability of nutrients required by the body as it is known to interfere with metabolic process so that growth and bioavailability of nutrients are adversely influenced [60]. However, the presence of antinutrients in edible plant species are normally low and below toxic levels [61]. For instance, the antinutritional properties such as oxalate, phytate, saponin, and tannin in sixteen wild underutilized fruits consumed in Nigeria show that the fruits analyzed were not up to the toxic levels of the antinutrients that can cause any adverse effect [60]. Furthermore, food processing methods such as soaking, germination, decortication, fermentation, and cooking influence both nutritional and antinutritional properties. Studies have shown that the presence of antinutritional factors in legumes reduced at varying degrees of food preparation methods [61]. More importantly, very high trypsin inhibitory activity in dry Indian bean reduced progressively by 51% after soaking for 12 hours, while tannins and phytic acids reduced to 46% and 36%, respectively [61]. On the other hand, other researchers have stated that the presence of antinutritional factors such as oxalates, phytates, and tannins which occur in varying amount in some plant species may be considered as a major barrier to the use; however, traditional methods employed in processing such as hydrothermal treatment, soaking, and fermentation reduce considerably the levels of antinutritional factors [62]. The aforementioned studies revealed that food to food fortification by using underutilized plant species could be challenged by antinutritional factors; however, the levels are not significant as well as the various preprocessing and processing techniques have shown the way to reduce its adverse effect. It is therefore very important to note that precooking treatments are vital when employing incorporation of underutilized plant species into food.

## 5. Conclusion and Way Forward

The potential benefits for fortifying staple food with indigenous underutilized edible crops have been investigated with promising advantages such as the following: it requires minimal infrastructure and involves simple technology for the ordinary rural household [63]. This is bright for alleviating under nutrition at the grass-root level. It could also lead to value addition of these crops and reduce postharvest losses via increased consumption. Most of these underutilized crops are indigenous, hardy, and able to withstand harsh environmental conditions; hence, it could be the solution to the impact of climate change on food security. More importantly, this type of fortification has proven useful for enhancing the nutritional value of most staple starchy foods in Africa. However, little attention is given to promote, implement, or evaluate the available technologies in community trials and in large-scale interventions that are striking [63]. Moving forward, rice meal fortified with UUPS should be investigated for curbing malnutrition and hidden hunger because rice is increasingly becoming the leading and important staple food in Africa. Additionally, it could be an effective vehicle to reach the majority of consumers. More so, it is recommended that the feed trial of the fortified staple foods with UUPS be researched to ascertain its direct impact on nutritional and health benefits instead of just analyzing the nutrittional content of staple food fortified with underutilized crops. Moreover, the health risk factors associated with antinutritional compounds and the lack of knowledge of the tolerance levels to these compounds in the human organism, the degree of variation of individual risks, and knowledge with respect to the influence of environmental factors on the detoxification capacity of the human organism [59] should be a concern.

## Figures and Tables

**Table 1:** Food to food fortification of staples and their nutritional benefits.

Staple foods	Underutilized potential food fortificants	Nutritional enhancements/benefits	References
Maize	Moringa oleifera	Improved the mineral composition and beta-carotene levels	[33]
	Soybean	Increased the protein content	[34]
	Common beans, cowpeas, and green peas	The formulation had enough energy and protein to meet the energy and protein requirement for 6-month infants	[35]
	Baobab fruit pulp, Moringa oleifera leaf	Increase calcium and iron contents of the meal	[16, 36, 37]
Cassava	Bambara flour	Enhanced protein and fat contents, and energy content increased in the composite flour	[38]
	Wheat and soybean	Improved consumer acceptability with increased protein content	[39]
	African yam bean, African breadfruit	Improved the protein content and consumer acceptability	[40]
	Pigeon pea flour	Protein and ash contents were improved	[41, 42]
	Soybean, melon seed, and Moringa oleifera seed flours	Protein, fat, and ash increased while carbohydrate decreased	[43]
Sweet potato	Avocado pear, Turkey berry	Improved the proximate and mineral contents	[32]
	Soybean and sorghum	Enhanced the protein, fat, and energy contents of the flour	[44]
	Bambara groundnut	Increased magnesium, phosphorous, potassium, and iron contents	[45]
	Moringa oleifera	Improved the lycopene, beta-carotene, protein, and fat contents	[46]
Yam	Soy, baobab pulp, and locust pulp	Improvement in the nutrient quality	[47]
	Moringa oleifera	Improved the nutritional value and influenced physicochemical properties (improving the textural characteristics a desirable characteristic of starchy meal)	[48]
	Cowpea	Enhanced fibre content and increased in vitro protein digestibility	[49]
Rice	Soybean, groundnuts	Improved the mineral, protein content, and dietary fibre of the cookies	[50]
	Soybean and groundnut	The diet was superior in terms of protein and energy content, sensory evaluation	[51]
	Soybean and wheat	15% level each would improve the nutritional quality without adversely affecting the sensory parameters	[52]
Sorghum	Pearl millet	Increased protein digestibility, soluble sugars, and mineral availability	[53]
	Baobab fruit pulp, Moringa leaf	Improved calcium, iron, and zinc	[16]
	Common beans, cowpeas, and green peas	High protein to meet the energy content	[35]
	Walnut and ginger	Increased the protein, fat content, and other useful properties	[54]
Cocoyam	Cowpea	Significant improvement in the chemical composition, including protein and micronutrients	[55]
Plantain	Moringa oleifera leaf powder	Protein, ash, and fat contents of the meal increased	[56]
Rice	Soybean and wheat	15% level each would improve the nutritional quality without adversely affecting the sensory parameters	[52]
	Soybean and groundnut	The diet was superior in terms of protein and energy content, sensory evaluation	[51]
	Soybean, groundnuts	Improved the mineral, protein content, and dietary fibre of the cookies	[50]
	Moringa oleifera	Better mineral content and high beta-carotene levels	[33]
Wheat	Quinoa, lupine, amaranth	Improved nutritional content in terms of protein and mineral composition	[57]
	Rice, soy bean, groundnut	Improved the mineral, protein content, and dietary fibre content	[50]
	Orange-fleshed sweet potato	Increased ash, fibre *β*-carotene, and quality of the bread	[58]
